# Community engagement matters: A call for greater advocacy in dermatology

**DOI:** 10.1016/j.ijwd.2021.01.008

**Published:** 2021-01-12

**Authors:** Alison Tran, Mona Gohara

**Affiliations:** a Frank H. Netter MD School of Medicine at Quinnipiac University, North Haven, CT, United States; b Department of Dermatology, Yale School of Medicine, Yale University, New Haven, CT, United States

**Keywords:** Dermatologic advocacy, Community engagement, Skin of color education, Self-esteem

The American Medical Association defines advocacy as a commitment “for the social, economic, educational, and political changes that ameliorate suffering and contribute to human well-being” (Earnest et al., 2010). The American Academy of Dermatology currently endorses advocacy priorities, including skin cancer prevention and access to treatment (among many others), with emphasis on teledermatology as a vehicle for access to dermatologic care. Teledermatology is beneficial for areas underserved by dermatologists and for constituents of vulnerable populations (Norton et al., 1997, Wong and Colven, 2019) because it overcomes barriers to time, transportation, distance, and mobility (Wong and Colven, 2019).

Furthermore, access to cellular and internet networks has outpaced access to health care in many nations, including low- and middle-income countries where medical access is limited (Wong and Colven, 2019). Although quality of care has reportedly improved throughout the years, access to care and health disparities have not (Buster et al., 2012). Although physicians, and our governing organizations, typically support the notion of advocacy as a professional responsibility (Earnest et al., 2010, Freeman, 2014), some evidence suggests that there is opportunity for more engagement (Freeman, 2014). Barriers to such initiatives might include, but are not limited to, a lack of exposure, time, or resources. We hope that sharing the details of our community project will help galvanize other dermatologists, encouraging them to share their expertise with populations outside of the office setting.

## #SkInterconnect

To reignite advocacy efforts locally, and in an endeavor to spread information on skin health in our community, we created a virtual workshop entitled #SkInterconnect, using the user-friendly platform Zoom. Our goal was to raise awareness about skin cancer and screening in communities of color. We aimed to engage inner city women of color in New Haven County during the COVID-19 pandemic.

Women comprise over half (52.7%) of the 130,250 people living in the New Haven area (U.S. Census Bureau, 2019). Black or African American, Hispanic or Latino, and Asian residents comprise 68% of this total population (not accounting for those who self-identify as mixed race/ethnicity). Close to 10% of the population age <65 years have no health insurance, and 25.9% live in poverty (U.S. Census Bureau, 2019). Although Connecticut is deemed one of the healthiest states in the nation, a report from January 2020 showed significant disparities in health by race and ethnicity that translated into poorer health outcomes, premature deaths, and an exorbitant amount of unnecessary health care costs (Connecticut Health Foundation, 2020). Our target audience was chosen based on these data.

In the span of 2 weeks, we reached out to the New Haven community to recruit local women of color in the low-income tier. Low income is defined as an annual income of ≤$26,535 in the state of Connecticut (Pew Research Center, 2018). Recruitment was through social media (Instagram, Twitter, and Facebook), direct e-mails to New Haven leaders (church/choir, dancers/choreographers, writers, radio station hosts), as well as the vice president for equity and inclusion at Quinnipiac University. The total participant group included eight women who identified as African American and one woman who identified as Indian American, with an annual income of <$26,535. Four women were between the ages of 15 and 24 years, and five women were between the ages of 25 and 34 years.

The content of the 1-hour workshop was centered on skin cancer screening/detection and prevention in skin of color, as well as the impact of hegemonic beauty on self-esteem and cultural/ethnic identity. As part of the event, we delivered a bag of generously donated hygienic and cosmetic products, including sunscreen from a small black-owned business, to each woman’s place of residence. The participants were invited to complete a 1- to 3-minute end-of-workshop survey for the purpose of quality improvement; 100% of participants voluntarily opted in. The survey also served as a vehicle to understand the impact of dermatologic advocacy and education in communities of color. This qualitative study was approved for institutional review board exemption under federal regulation.

All participants strongly agreed with the following statements: “The workshop increased my understanding about the impact of hegemonic beauty on self-esteem in women of color” and “I feel like I can share what I learned today with my friends, family, and/or community members.” Eight participants strongly agreed that the workshop increased their understanding of cancer in skin of color and wanted to see more physicians and healthcare professionals involved in the community (Table 1), and narratives were offered by several participants (Table 2).

**Table 1:** Summary of survey responses.

	Respondents, n (%)
**“This workshop deepened my understanding about skin cancer (including screening and prevention) in skin of color.”**	
Agree	1 (11.11)
Strongly agree	8 (88.89)
**“The workshop increased my understanding about the impact of hegemonic beauty on self-esteem in women of color.”**	
Strongly agree	9 (100)
**“I feel like I can share what I learned today with my friends, family, and/or community members.“**	
Strongly agree	9 (100)
**“I foresee myself participating in more workshops like this in the future.”**	
Agree	1 (11.11)
Strongly agree	8 (88.89)
**“I would like to see more physicians and health care professionals involved in my community.”**	
Agree	1 (11.11)
Strongly agree	8 (88.89)
**“The overall quality of this workshop was excellent.“**	
Agree	1 (11.11)
Strongly agree	8 (88.89)

**Table 2:** Participant narratives.

“I wholeheartedly enjoyed this workshop. It was informative without being too academic. Both speakers were relatable, warm, and engaging. The workshop proved there is undoubtedly a need for diverse advocacy in dermatology. Learning about proper skin protection as people of color during the workshop led me to wanting to know more about proper skin care as a person of color-- and skin all over the body, especially the parts that aren't often exposed. Overall, it was definitely a worthwhile workshop with invaluable information that I am grateful to have been a part of! I look forward to the next one!”
“This workshop was very informative and enlightening, living in proximity to areas of great cultural diversity and economic diversity, more people should be educated about these topics.”
“This was very informative and both speakers were amazing! They spoke to us as if they were our friends and that helped with the connection and understanding information better!”
“I loved being a part of it.”

This initiative can serve to highlight the importance of local engagement and interconnection with communities of color while eliminating some health care barriers. This type of workshop can be easily emulated, has potential for program scalability, and requires minimal resources except for valuable time and knowledge. Residency programs and dermatology interest group members can readily facilitate similar programs, particularly as the number of diversity and outreach task force committees increases. The concept of televolunteerism can be an annual or semi-annual option during training. Also, there is opportunity for collaboration among dermatologists across many geographic regions to facilitate workshops similar to #SkInterconnect. This type of aggregate advocacy can be a conduit to reach patients in rural, poor areas that are densely populated with minorities who may have inadequate access to dermatologists. Of note, most counties with African, Hispanic, and Native American majorities have no dermatologists (Vaidya et al., 2018). Also, minority physicians are more likely to care for minority patients and practice in underserved areas (Vaidya et al., 2018). Virtual initiatives could readily be a first step in broadening the scope of patients seen by health care providers.

Through advocacy, health inequities can be addressed to improve the health of patients in the context of the community and population. In agreement with Earnest et al. (2010), advocacy should not be a parochial concern if it is a core element of professionalism. Health advocacy should extend beyond changes to health behavior or health interventions and instead address systemic issues that drive disproportionate health outcomes. This liberates individuals from blame and avoids assuming that individuals have the ability or resources to make health-informed decisions (Earnest et al., 2010). According to Freeman (2014), physicians need not resort to “simply patching up” the sick, but rather fight for the prevention of sickness. Physician health advocates not only understand the upstream factors that engender health inequities, but recognize the impact of systemic inequities beyond the patient. Health care disparities in dermatology clearly exist; thus, it is important that dermatologists project a unified voice regarding this matter because even though dermatologists represent <2% of all physicians, they “successfully project a voice much larger than their numbers” (Elston, 2014).

## Conflicts of interest

None.

## Funding

None.

## Study approval

The author(s) confirm that any aspect of the work covered in this manuscript that has involved human patients has been conducted with the ethical approval of all relevant bodies.
